# A Model for Creating a Supportive Trauma-Informed Culture for Children in Preschool Settings

**DOI:** 10.1007/s10826-014-9968-6

**Published:** 2014-05-20

**Authors:** Cheryl Holmes, Michelle Levy, Avis Smith, Susan Pinne, Paula Neese

**Affiliations:** 1 School of Social Welfare, University of Kansas, Lawrence, KS USA; 2 Crittenton Children’s Center, Kansas City, MO 64134 USA

**Keywords:** Early childhood trauma, Intervention, Early childhood mental health, Head Start, Classroom-based consultation

## Abstract

The all too common exposure of young children to traumatic situations and the life-long consequences that can result underscore the need for effective, developmentally appropriate interventions that address complex trauma. This paper describes Head Start Trauma Smart (HSTS), an early education/mental health cross-systems partnership designed to work within the child’s natural setting—in this case, Head Start classrooms. The goal of HSTS is to decrease the stress of chronic trauma, foster age-appropriate social and cognitive development, and create an integrated, trauma-informed culture for young children, parents, and staff. Created from a community perspective, the HSTS program emphasizes tools and skills that can be applied in everyday settings, thereby providing resources to address current and future trauma. Program evaluation findings indicate preliminary support for both the need for identification and intervention and the potential to positively impact key outcomes.

## Introduction

Exposure to potentially traumatic events is an all too common experience for many children, including those who are preschool-aged. In a national sample, Finkelhor et al. (2009) found that more than 60 % of children aged 0–17 experienced or witnessed victimization such as child maltreatment, bullying, or assault within the past year. Specific to younger children, a recent study exploring the prevalence of trauma exposure found that by their forty-eighth month, one in four children had experienced or witnessed an event that could be deemed potentially traumatic (Briggs-Gowan et al. 2010). Likewise, a study of 155 Head Start participants found 78 % of the children’s self reports and 66 % of parent reports indicated exposure to at least one incident of community violence (Shahinfar et al. 2000). Community violence in this study included incidents such as beating, shooting, stabbing, or robbery where the child was a victim or witness. Minority children in inner-city environments are particularly vulnerable to experiencing trauma due to high community rates of poverty, drug use, and crime (Stein et al. 2003; Ghosh Ippen et al. 2011). The range of events that young children may experience as traumatic is potentially broadened by the natural limitations in a young child’s capacity for self-protection.

Researchers have linked trauma exposure to a broad spectrum of difficulties with socio-emotional development in preschool children that includes impairment in attachment, biology, affect regulation, dissociation, behavior regulation, cognition, and self-concept (Lieberman et al. 2011; Spinazzola et al. 2005; Cook et al. 2005). Early traumatic experiences have been associated with both internalizing (e.g., depression, anxiety) and externalizing (e.g., aggression) symptoms (Ghosh Ippen et al. 2011). Children impacted by complex trauma, defined as exposure to multiple or chronic traumatic events typically early in life, are particularly vulnerable to negative effects (Spinazzola et al. 2005). Traumatic life events experienced in early childhood can result in a wide array of adverse outcomes that may extend well into adulthood, such as alcoholism, depression, poor self-rated health, and diseases like cancer (Felitti et al. 1998). Despite these potentially substantial impacts, many traumatized young children may not be identified as such and are not often found in mental health systems (Graham-Bermann et al. 2012; Lieberman et al. 2011). However, young children and their families are often connected with other community services such as child care, early education/Head Start, and pediatricians among others which offer effective avenues for extending mental health consultation and services, as well as raising awareness on the effects of trauma and need for mental health supports (Lieberman et al. 2011; Osofsky and Lieberman 2011).The wide range of behaviors exhibited by young children impacted by trauma can present challenges in an early childhood education setting as effects may be seen in multiple domains: affective, behavioral, physiological, and relational (Lieberman et al. 2011; Cook et al. 2005). For some children, the trauma can lead to cognitive distortions which translate into thoughts like: the world is not safe, I am not good enough, or things will never get better. They try to cope with these feelings and respond appropriately to their environment, but often, in the absence of specific treatment, they may develop physical pain (e.g., stomach ache, headache, etc.) or may display aggressive behaviors such as tantrums, verbal abusiveness, or hitting. Unfortunately, externalizing behaviors in particular affect not only the child but also the teacher and the larger classroom (Blodgett 2012). Despite their young age, these behaviors can lead to negative ramifications including expulsion from the pre-kindergarten setting. Gilliam (2005) found that children in pre-kindergarten programs have expulsion rates three times higher than youth in K-12. Rates of expulsion were highest for boys, African-American and older preschoolers.

Children affected by trauma need a safe, caring, and consistent environment (Swick et al. 2013). Preschool programs, such as Head Start, provide an ideal setting through which to identify these children and provide early on-site treatment and prevention (Bratton et al. 2012). The impact of trauma on developmental trajectories and school readiness produces an impetus for Head Start programs to play a role in early identification and intervention (Garro et al. 2011; Lieberman et al. 2011). This article reports on the development of a model to create a trauma-informed culture to meet the needs of young children in a preschool setting.

### The Advent of Head Start Trauma Smart

It is within the context of pervasive trauma and a significant desire to address its effects among young children that a mental health provider (Crittenton Children's Center) came together with local Head Start programs to conceptualize, develop, and implement Head Start Trauma Smart (HSTS). In 2007, HSTS staff members who provided mental health services to the local Head Start community noticed there were large numbers of funerals occurring related to Head Start families and staff. Further investigation showed that between 2004 and 2007, there were 40 deaths as a result of interpersonal violence, accidents, untreated health problems, and other potential trauma-producing situations. Although young children are greatly affected by exposure to violence and loss, HSTS staff noticed that there was limited knowledge and recognition about the impact of trauma on young children.

With this backdrop, HSTS staff began to pursue trauma-specific supports for children ages 3–5 and their families. The goal was to have a multi-faceted approach that utilized evidence-based or evidence-informed practices for the intervention, a model that would involve the entire organization serving the child, and one that would not risk re-traumatizing the child. HSTS’s search of resources revealed that existing trauma responses were generally designed for older children and that researchers were still learning about effective intervention with preschool children impacted by complex trauma exposure. After engaging local Head Start partners, exploring various approaches, and talking with trauma leaders and specialists throughout the country, HSTS chose to integrate three existing evidence-informed modalities to create the unique approach of HSTS, modifying components as needed so they would be appropriate for young children. These three modalities are described below.

### Training Based on the Attachment, Self Regulation, and Competency (ARC) Model

**Fig. 1:**
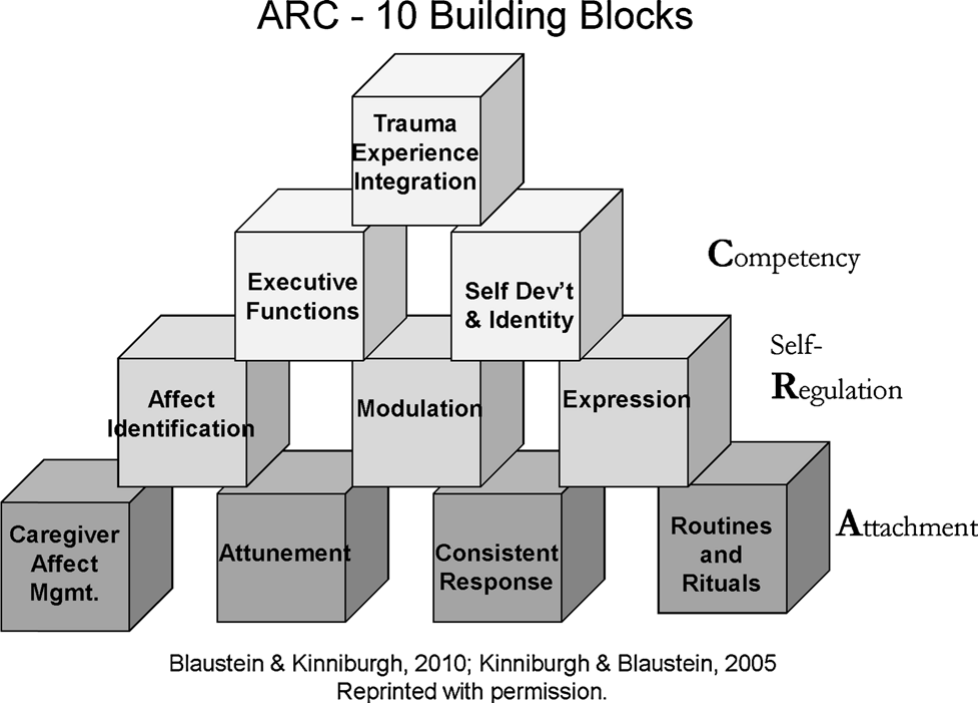
10 Building blocks for the three ARC core domains

The Attachment, Self Regulation, and Competency (ARC) framework is a complex trauma-focused intervention/model developed by Blaustein and Kinniburgh (2010) at the Trauma Center at the Justice Resource Institute in Brookline, Massachusetts. It outlines three core domains impacted by exposure to chronic, interpersonal trauma: attachment, self-regulation, and developmental competencies. Within those domains are ten core building blocks of intervention meant to translate across service settings and service delivery format, including non-traditional clinical settings (see Fig. 1). Recent preliminary research shows that the ARC model may hold some promise in improving clinical outcomes for young children exposed to a wide range of traumas (Arvidson et al. 2011). A small sample of children receiving outpatient services based on the ARC framework showed a 19-point improvement on Child Behavior Checklist (CBCL) scores compared to a 2.5-point improvement for those who did not complete treatment. The authors do not specify whether this is a statistically significant difference. ARC is listed on the Empirically Supported Treatments and Promising Practices page on the National Child Traumatic Stress Network website. Specific details about the ARC framework provided in 2012 can be found at http://nctsn.org/sites/default/files/assets/pdfs/arc_general.pdf.

### Trauma-Focused Cognitive Behavioral Therapy (TF-CBT)

Trauma-focused cognitive behavioral therapy (TF-CBT) consists of a set of trauma-specific components that includes: psychoeducation, parenting skills, relaxation skills, affective modulation, cognitive coping, trauma narration and processing, invivo mastery, conjoint parent–child sessions, and enhancing safety (Cohen et al. 2006). In a comprehensive review of evidence-based psychosocial treatments for children exposed to traumatic events, TF-CBT was the only treatment identified as *well*-*established* to significantly improve symptoms of post-traumatic stress disorder for children and youth (Amaya-Jackson and DeRosa 2007; Silverman et al. 2008). Young children can cooperate meaningfully in cognitive-based therapy that addresses trauma (Scheeringa et al. 2007). Yet only two randomized controlled trials of TF-CBT involving preschool children have been reported and both focused narrowly on children who had been sexually abused (Stallard 2006).

### Early Childhood Mental Health Consultation

Early childhood mental health consultation typically involves a mental health clinician working within a preschool setting to decrease problem behavior and promotes socio-emotional development through facilitating changes in teacher behavior and the classroom environment. In a recent systemic review, mental health consultation was consistently associated with a decrease in externalizing behaviors and increase in prosocial behaviors in preschool children (Perry et al. 2010). The review also found that mental health consultation was associated with teachers’ self-reported increases in competencies and effectiveness. In Gilliam’s (2005) study on expulsion rates among preschoolers, the likelihood of expulsion significantly decreased with access to classroom-based mental health consultation. The studies cited did not measure whether the children were exposed to trauma.

### HSTS Program Description

The purpose of HSTS is to decrease the stress of chronic trauma, foster age-appropriate social and cognitive development, and create an integrated, trauma-informed culture for young children, parents, and staff. Utilizing the above modalities, the HSTS program is comprised of four components described below:

1. *Training* is offered by HSTS therapists to the various people who touch the life of the child, including Head Start staff in all positions (e.g., administrators, receptionists, bus drivers, teachers, etc.), the child’s parents, and the child’s broader network: close neighbors, grandparents, and informal day care providers. The therapists are master’s level, licensed clinicians with a preference given to those with a trauma-informed background and/or early childhood training.
  The training framework is based on the 3 domains and 10 building blocks of ARC, although specifically adapted for early childhood use. For example, for the Affect Identification block, adults learn to show children pictures of people with different expressions, help the child to identify the person’s feelings, and then work with the child to identify how he or she is feeling. To help children modulate their feelings, adults learn how to teach children to take a deep breath using different props and then to remind the children to take a deep breath when they have a “big feeling”. The intent is to have tools and skills that any adult can use, thus allowing a trauma-informed environment to be integrated in all parts of the child’s life.
  Adaptions to ARC for HSTS fall into three areas, although no modifications have been made to the ARC framework or its conceptual content. First, the ARC concepts have been translated into terms for a non-clinical, lay audience. Second, additional age-appropriate resources have been created specifically for HSTS and its target population of children ages 3–5 and their caretakers, which are then shared and used in training and therapy. Examples of the hands-on tools include props and games to help develop attachment, self regulation, and age-appropriate developmental competencies. More broadly, the training is designed to help participants become more aware of the need for early identification of children who may need trauma support and to teach the use of developmentally appropriate, hands-on tools that teachers, parents, children, and others can easily integrate into the child’s everyday environment. Through exposure to and use of the training information and tools, adult participants are able to think about how a child’s behavior aligns with the three ARC domains and related building blocks and how the behavior is likely a superficial reaction to what is happening at a deeper level. With this understanding, adults are better able to slow down and look beyond a specific behavior. Finally, adaptations have been made to the training format, which is often done over two 6-h days. Specifically, HSTS typically delivers the ARC training in ten 2-h sessions, covering one ARC building block at a time. This format tends to work better for Head Start staff and parents and allows them time to try out the hands-on tools during the training and then practice the new skills between sessions.
2. *Intensive Individual Trauma-Focused Intervention* is available for referred children who meet criteria based on the Achenbach Teacher Report Form/CBCL and Childhood Trust Events Survey results. Staff or parents can refer a child to HSTS who may have witnessed or experienced trauma or who they feel might benefit from intensive, individual services due to internalizing/externalizing behaviors. Services are provided by masters-level therapists with degrees in social work or counseling who either have or are working on clinical licensure. Staff has been trained in trauma-focused treatment models including ARC and TF-CBT, both of which have been adapted to align developmentally for 3–5 year olds. Similar to the adaptations described above for ARC, changes to TF-CBT involve modifications to align with the developmental needs of 3–5 year olds and the realities of parents with young children who are living in poverty. First, there are generally more sessions of shorter duration–around 30–45 min for 12–24 sessions–and may include strategies like play therapy, bibliotherapy and a sand tray along with focusing on cognitive distortions more so than the trauma narrative. Second, parent involvement is still highly encouraged and desired and parents do participate to the extent that they can. However, many of the families face transportation difficulties and may work multiple jobs that include daytime, evening, and overnight shifts, making it difficult for them to participate in their child’s weekly session. Therefore, therapists make weekly phone calls and send notes to the parents after each session, detailing the session content and providing concrete ideas that parents can use at home to reinforce therapeutic concepts. The therapists can make home visits in addition to providing the therapy at the Head Start.
3. *Classroom Consultation* is provided by HSTS therapists to all teachers and children as requested or as needed, regardless of whether or not a child in the classroom is receiving intensive individual treatment. Overall, the consultation time allows the therapist to bring the skill-based training into the classroom and support the teacher in implementing what was learned. During the consultation time, the HSTS therapist shares resources and ideas that have primarily been developed by HSTS staff based on ARC principles or TF-CBT. They also help set up the classroom to create a supportive trauma-informed environment (e.g., adding a calm down area with child-friendly resources that children can access when they feel they need to).
4. *Peer Based Mentoring* is a more recent addition and was created to help address consistency and sustainability of the other components. Staff Peer Mentoring offers a way for teachers and supervisors to support each other and talk about the techniques and skills being used. Development of a similar Parent Peer Mentoring is now underway. Both programs are intended to help program participants continue using and implementing the skills.

**Fig. 2:**
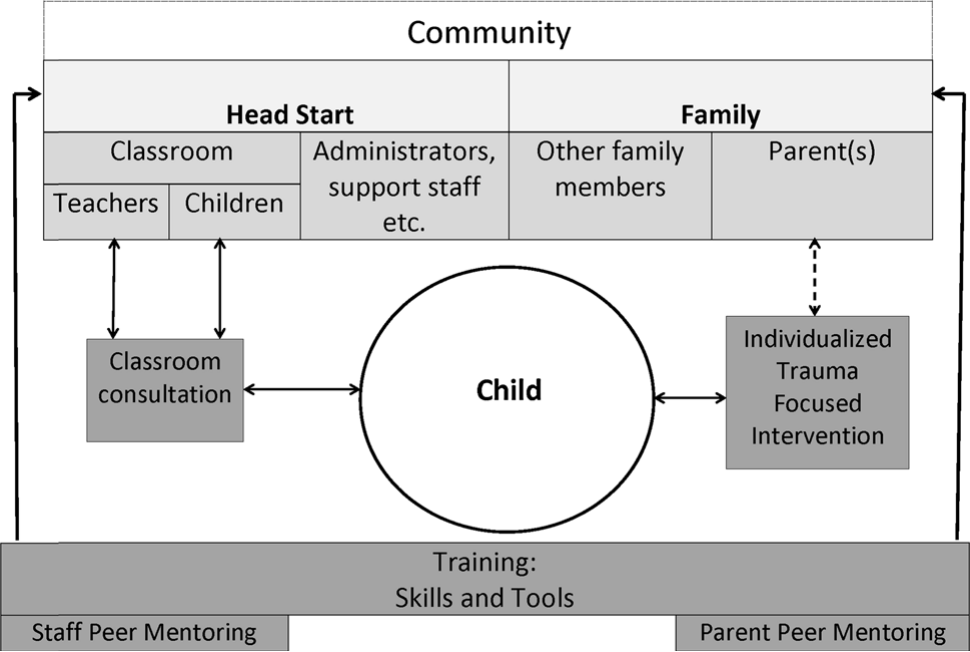
Conceptual overview of Head Start Trauma Smart

Figure 2 provides a conceptual picture of HSTS, with the child in the center and the training serving as a core foundation. The dotted line between parent and intervention indicates encouragement for the parent to be involved as much as possible. While the child is clearly central to this work and is the primary client, parents, teachers, and the larger group of children at participating Head Start programs are also beneficiaries.

This type of integrated and applied model to create a trauma-informed environment is needed for several reasons. First, it focuses on developing skills and a consistent plan of action for caretakers (teachers and parents) who are in frequent contact with the children. Thus, a child in need does not have to wait for time with a therapist to receive trauma-based support. Second, experience has shown that adults can have untreated traumatic situations that can be triggered by the child’s situation. Parents may also experience secondary trauma as a result of the child’s trauma. Blodgett (2012) notes that trauma (or “adversity”) is common among Head Start children and parents. Therefore, they can apply the skills and training to themselves. And third, casting a wide net to teach basic skills that can be used by lay people in everyday situations—adults and children alike—can help build resiliency for future situations.

### HSTS in Practice

Although HSTS has expanded to additional locations, information presented here will focus on implementation in three Midwestern urban inner-city Head Start programs. Across these three programs, there are more than 400 Head Start staff members who serve almost 1,100 children ages 3–5. Overall, approximately 60 % of the children in these three programs are African-American, 30 % Hispanic, 6 % Caucasian, and 4 % other.

To begin, HSTS training was provided to the Head Start teachers, administrators, receptionists, bus drivers, and kitchen staff. At smaller sites, all staff were trained during the first year. Larger sites required 2 years for everyone to receive training. The training covered the 3 domains and 10 building blocks of ARC and included time to learn and practice with developmentally appropriate hands-on tools created by HSTS to align with the building blocks. The training typically occurred in ten 2-h sessions over multiple months so that all 10 building blocks would be covered with time to practice in between sessions. In subsequent years, newly hired staff received the full HSTS training and previously trained staff received a booster session that ranged from 2 to 6 h depending on agency needs. Through increasing skills and knowledge of participants, this training is designed to facilitate systemic changes by creating a trauma-informed culture that positively impacts all children.

Children were identified for referral to the program by either the child’s teacher or parent, and typically, although not always, were referred due to the child’s externalizing behaviors. Upon referral, both the parent and the teacher completed their version of the Achenbach Teacher Report Form/CBCL. The parent also filled out the Childhood Trust Events Survey on behalf of the child. The HSTS therapist reviewed these completed forms, met with the child, observed him/her in the classroom and completed additional diagnostic information. There were several possible outcomes to the referral including the following:

If the child had exposure to multiple or chronic trauma as indicated by the Childhood Trust Events Survey and had scores in the clinical range on the Achenbach Teacher Report Form/CBCL instrument, he/she qualified for intensive individual trauma-focused intervention. Classroom consultation was also available to these children. Parents of these children were encouraged to attend therapy sessions to the extent possible, meet with therapists for psycho-education, and review weekly written (and often verbal) updates about their children’s progress. If the child did not have an identified traumatic event, but still had scores in the clinical range of concern, staff provided individualized interventions for the child, such as play therapy, and psycho-educational support for teachers and parents.

If the child had an identified traumatic event but did not have scores in the clinical range, staff provided psycho-educational support for the parents and teachers so they could be aware of signs and symptoms in the event these issues surfaced at a later date. This support was tailored to the child and involved the use of ideas from the National Child Traumatic Stress Network and books related to the child’s situation that the parents and teachers could use. The HSTS training was also available to them to help them understand trauma as it relates to young children.

If the child had not experienced a traumatic event and did not have scores in the clinical range, they did not qualify for trauma-based intensive treatment. However, teacher, parent, and child consultation could still be provided to help address the specific reasons the child was initially identified and referred. It is anticipated that all children can benefit from consultation as it supports teachers to integrate HSTS techniques into the classroom.

## Method

From the outset, HSTS was intended to be a practical approach for working with children, families, and providers; it was not created as a research study. However, HSTS staff did identify and utilize standardized instruments from the beginning in order to measure systemic improvements in the classroom (as measured by CLASS scores) and whether the children who were receiving individual treatment were making clinical progress (as measured by the Achenbach CBCL and TRF 1.5–5). An external independent evaluation and related data analyses and reports were conducted by Resource Development Institute (RDI) through a contract with HSTS from the onset of the project. RDI sent draft versions of the evaluation reports to HSTS prior to finalization but this was only to allow HSTS to provide input regarding programmatic accuracy. The data summaries were in PDF and were not available for editing.

An overview of instruments used for data highlighted in this article follows. Other program data not reported here are collected at regular intervals, including satisfaction surveys with administrators, teachers, and parents.

### Measures

Table 1 provides an overview of measures for data and the timing of each. A description of each measure follows the table.

**Table 1:** Overview of measures used for HSTS

Instrument	Timing	Informant
Childhood Trust Events Survey	Completed at time of HSTS referral for individual treatment	Parent/guardian
Achenbach–Teacher Report Form and Child Behavior Checklist (CBCL) (version nationally normed for 1.5–5 years)	Completed at time of referral and either when treatment ends or every 6 months, whichever comes first	Teacher Parent/guardian Other caregivers as needed
Classroom Assessment Scoring System (CLASS)	Observations are done in all classrooms prior to staff training and then again later in the same year and twice in each subsequent year	HSTS therapists or Head Start staff but must be a certified CLASS observer

#### Childhood Trust Events Survey (CTES): Caregiver Version

This questionnaire was developed by individuals at the Cincinnati Children’s Hospital Medical Center–Trauma Treatment Training Center. It is intended as a screening instrument to capture historical information, not as a diagnostic tool. As such, psychometric properties are not applicable (Olafson and Connelly 2012). The CTES–Caregiver Version is designed to be completed by the parent or caregiver for children under age eight. It contains 26 items about specific traumatic events across a range of topics such as accidents, abuse, other types of violence, serious medical situations, and loss of caregivers (death, prison), among others. Response options are yes, no, or don’t know. Some of the survey items are borrowed from the UCLA PTSD Index and the Traumatic Events Screening Inventory for Children. All items from the Adverse Childhood Experiences are also included in the CTES.

In the HSTS program, parents are asked to complete this survey at the time the child is referred for HSTS assessment. Completed information is returned to the HSTS therapist. The purpose is to help determine need for intensive services and, when need is identified, to help tailor the therapy to the child’s trauma experience. Information about CTES and a copy of the instrument are available at http://www.ohiocando4kids.org/Childhood_Trauma.

#### Achenbach System of Empirically Based Assessment

The Achenbach is a diagnostic tool that assesses child behavior as aligned with the DSM and has good reliability and validity (Achenbach and Rescorla 2000). HSTS uses the versions that are normed for children ages 1.5–5 to assess clinical changes over time. Results include Internalizing, Externalizing, and Total Problem scores along with scores for syndrome scales. The parent completes the CBCL and the teacher completes the Teacher Report Form.

#### Classroom Assessment Scoring System (CLASS)

This instrument, now used by the Federal Office of Head Start, assesses quality of relationships in the classroom environment (adult to adult, adult to child, and child to child). The CLASS has three domains: Emotional Support, Classroom Organization, and Instructional Support. Within those areas, there are 10 dimensions. Scores range from 1 (low) to 7 (high). This instrument has been found to have good reliability and validity (Teachstone, n.d.). CLASS scores relate to the classroom, not individual children. HSTS therapists are certified observers with the CLASS using it to assess potential impact of HSTS in the classroom and to help the Head Start agencies achieve their CLASS threshold targets.

### Participants

Roughly 150 children were referred for assessment for HSTS intensive services during the 2011–2012 school year. Over half of these children (n = 81) were found to need therapeutic intervention based on the CTES and the Achenbach. These children were more likely to be male (64 %), ranged in age from 31 to 76 months and had a mean age of 4.25. Race/ethnicity data showed 39 % were African-American, 15 % non-Latino white, 8 % Latino/Latina, 3 % other, and data unavailable for about one-third (35 %).

## Results

Highlights from the three data sources are shown below: Childhood Trust Events Survey for Caregivers (CTES), Achenbach (Teacher Report and CBCL/Parent), and CLASS.

Data for the CTES and Achenbach are for the same group of 81 children but there were eight CTES instruments that were not completed by the parent/guardian which accounts for the difference in number between the two sets of data. Children in this group of 81 received the following components: intensive trauma-focused intervention which involved approximately 12–24 weekly sessions of 30–45 min as well as approximately 6 h a month of classroom consultation.

Results from the CTES showed that 74 % of the caregivers reported that their child had been exposed to at least one traumatic event, 60 % reported at least two traumatic events, and close to one-half (45 %) reported exposure to three or more traumatic events. Table 2 shows the eight events from the 26 CTES survey item list that were most often selected.

**Table 2:** Most frequently reported type of trauma events for HSTS children

Event	% That chose “yes”^a^
Has your child ever had a family member who was put in jail or prison or taken away by the police?	41
Was your child ever completely separated from his/her parent(s) for a long time, such as going to a foster home, the parent living far apart from him/her, or never seeing the parent again?	32
Has your child ever had a family member or someone else very close to him/her die unexpectedly?	26
Has your child ever had someone living in his/her home who abused alcohol or used street drugs?	23
Has your child ever seen or heard a family members being hit, punched, kicked very hard, or killed?	22
Has your child ever seen or heard family members act like they were going to kill or hurt each other badly, even if they didn’t actually do it?	19
Was your child ever so badly hurt or sick that he/she had to have painful or frightening medical treatment?	19
Has your child ever had a family member who was depressed or mentally ill for a long time?	19

From Resource Development Institute (2012a, July, p. 3). Adapted with permission. Items displayed are the top one-third events selected by caregivers using The Childhood Trust Events Survey Children and Adolescents: Caregiver Form

^a^Percentages based on 73 caregivers reporting

For those children who received intensive services from the HSTS program, results from the Teacher Report Form of the Achenbach noted statistically significant changes in four areas that are particularly important for school readiness and overall academic performance. A paired-samples *t* test was conducted to determine whether or not the pre- and post-test scores were significantly different from each other while determining the probability of a Type 1 error. Specifically, improvements were seen in the ability to pay attention, which is an important ability for receiving classroom instruction. Improvements were also seen in externalizing behavior and oppositional defiance. If left untreated, these can potentially lead to suspension or expulsion in extreme cases (Table 3).

**Table 3:** Pre/post mean scores for HSTS children receiving intensive treatment

Scale	Pre mean t-score	Post mean t-score
Attention problems	62.50	59.93*
Externalizing problems	63.37	60.89*
Attention deficit/hyperactivity problems	63.30	60.60*
Oppositional defiant problems	65.42	62.89*

From Resource Development Institute (2012a, July, p. 5). Adapted with permission. Table displays items that had statistically significant changes between time of referral and termination from intervention or end of the school year, whichever came first, using Achenbach Teacher Report Form. n = 81

* *p* < .05

In addition to teacher reports, parents completed the Parent CBCL. Although the number of parents completing the Achenbach was lower (n = 42), overall numbers supported the general positive trend reported by teachers in key outcomes that can affect school experience and readiness. Similar to teachers, t-scores showed that parents reported significant improvements (*p* < .05) in externalizing problems and attention/hyperactivity. They also showed that parents reported a significant improvement (*p* < .05) in internalizing behaviors as well, moving from a pre mean score of 54.57 to a post mean score of 50.91 (Resource Development Institute 2012a, July, p. 5).

Table 4 shows changes in CLASS scores over time on a scale of 1–7. CLASS scores relate to the classroom, not individual children, and are designed to reflect the quality of the relationships in the classroom (adult to adult, adult to child, and child to child). While obtaining a seven is the long-term outcome for each domain or dimension, with the exception of Negative Classroom Climate where the best score is a one, the short term goal is to have steady progress in moving toward the goal. The RDI report (2012b, December, p. 7–8) notes that statistical significance could not be calculated for CLASS scores because of turnover and tracking issues between the years. However, the overall trend for all categories of CLASS scores over a 2 year period is moving in the desired direction.

**Table 4:** Mean scores for quality of relationships in HSTS-Head Start classrooms

Domain	Dimension	Mean score		
		Oct. 2010	Oct. 2011	Oct. 2012
Emotional support domain^a^		4.60	4.92	5.33
	Positive classroom climate^a^	4.56	5.01	5.59
	Negative classroom climate^b^	1.76	1.50	1.39
	Teacher sensitivity^a^	4.00	4.15	4.67
	Respect for student perspective^a^	3.59	4.04	4.44
Classroom organization domain^a^		4.02	4.65	4.54
	Behavior management^a^	4.20	4.88	4.78
	Productivity^a^	4.58	5.32	5.00
	Instructional learning formats^a^	3.29	3.75	3.83
Instructional support domain^a^		2.17	1.71	2.35
	Concept development^a^	1.76	1.32	1.90
	Quality of feedback^a^	2.18	1.70	2.55
	Language modeling^a^	2.58	2.10	2.61

From Resource Development Institute (2012b, December, p. 30). Adapted with permission. Domain Areas are from the CLASS instrument. n = 60 classrooms

^a^Seeking increase on scale of 1–7

^b^Seeking decrease on scale of 1–7

## Discussion

HSTS represents an innovative integration of evidence-informed modalities for the purposes of creating a developmentally appropriate intervention to address complex trauma among young children. HSTS is unique in its attempt to develop a trauma-informed culture among the multiple caregivers (parents, teachers, and others) who influence a child’s development. While much remains to be known about effective interventions for this population, the HSTS model offers an approach that deserves further study.

The genesis of HSTS grew out of the perception that the young children in the preschool classrooms were exposed to traumatic events. Data collected through the Childhood Trust Events Survey supports other research showing that many preschool children are indeed exposed to these types of situations (Briggs-Gowan et al. 2010; Graham-Bermann et al. 2012; Shahinfar et al. 2000). Numbers are likely below actual rates of need as there is indication that the impact of trauma exposure may be under-identified in preschool children due to the misperception that young children are not affected in the same way by trauma and because there continues to be stigma around asking for help (Lieberman et al. 2011; Lieberman and VanHorn 2009). Given what we know about prevalence of trauma in young children, its impact on their learning and development, and the long-lasting effects it can have, it is critical that intervention occurs as early as possible to mitigate these negative results.

While evidence-informed modalities exist for serving young children and their families, they tend to focus on a specific approach—training, classroom consultation, or intensive therapy. However, a child’s life is not segmented and does not mirror a controlled laboratory environment. There is a need to have a model that can fit into the myriad of natural settings (home, classroom, etc.) that create the child’s environment and have applied tools that can be shared across the various caregivers—parent, teacher, therapist, and administrators, among others. This is why HSTS integrates three evidence-informed modalities to create a model that includes training, classroom consultation, intensive therapy, and peer mentoring.

For the children in need of intensive therapy, this approach that includes multiple points of intervention allows the child to receive a consistent, repetitive message from the therapist, teacher, and parent so it can then be internalized. This approach also builds the skill set of the caregivers and encourages them to have resources readily available in the classroom or home which creates a larger, trauma-informed culture. Thus, the impact of HSTS goes beyond children receiving intensive services and helps prepare children and adults who may experience trauma at a later date.

Rigorous research is still needed, but the preliminary HSTS results are quite encouraging. Significant changes were seen in the teacher report of key externalizing outcomes that can have implications in the classroom. Parents noted positive changes in both internalizing and externalizing behaviors. Parents, teachers, and administrators generally reported satisfaction with the HSTS program.

Although HSTS was initially implemented in an inner-city community, trauma is not limited to urban environments or to a specific sub-population. And, while all Head Start programs have some similarities based on federal requirements, each one is tailored to address and to reflect the unique needs, resources, and culture of its community, agency, staff, and families. HSTS was designed in the field and, therefore, has already created mechanisms for allowing some tailoring in order to fit the environment. During design and initial implementation, HSTS staff worked with local Head Start administrative leaders and staff who willingly shared their knowledge, experiences, and insights to identify the practical realities for HSTS. HSTS developed local agency advisory boards with members from all areas of Head Start (administration, teaching staff, ancillary staff, and parents). The board members contributed ideas for planning, designing, measuring, assessing, evaluating, and improving services throughout the project. HSTS also developed a metro-wide advisory board so that agencies could learn and grow from interactions with each other. This participation resulted in critical information about what likely would or would not work generally as well as where programmatic flexibility would be required among the different sites.

HSTS has also been created as a complementary, not a stand-alone, program so it can be used along with other social-emotional curricula that may be in use. Overall, the HSTS program strives to balance fidelity to the model with flexibility in implementation so that it can meet varying cultures and needs. HSTS is now being used in non-urban settings to explore how the intervention will work in settings with different resource availability and cultures.

While results are encouraging, limitations are present. The data collected are on children referred and served. No control group has been used to date. Also, due to the newness of the design and intervention, the model does not have a specific fidelity against which implementation is measured. Finally, data only reflects use with children in an urban inner core setting. It is not yet clear how HSTS will work in a rural setting.

HSTS has initiated some program innovations to address these limitations. A peer mentoring component has been created to help staff and parents support each other in key aspects of the intervention. Replication is currently happening in more rural settings. HSTS staff members are exploring sustainability questions, including the intervention of the model in the event that specially trained therapists are not available and further defining the model so that fidelity measures can be created.

Implications for future research are related to the topics previously mentioned. Specifically, there is a need to test the impact of components independently and with a control group. There is also a need to follow children/parents longitudinally to see if gains are sustained over time. Research on the impact in the classroom with and without booster sessions for teachers could yield interesting information as would information on the extent to which teacher knowledge and skills are changing specific to trauma. Related to the Achenbach scores (Teacher Report Form and Parent CBCL), there are times when scores for individual children show clinical improvement (e.g., moving from the clinically significant range to borderline or normal range) yet, the mean score for the group does not show statistically significant change. Further study of how often this occurs and in which areas could be informative. Finally, given that parents, teachers, and caregivers might also be impacted by trauma, studying the effects of training on these participants could yield valuable information.

## Conclusion

There is a clear need for applied evidence-informed interventions and trainings to help young children who have experienced traumatic situations and those who care for them. Yet, few developmentally appropriate options exist, particularly when the goal is not only to address the specific child in need but also to create an overall trauma-informed model that can help build the resiliency of the larger community. HSTS offers a promising developmentally appropriate solution that combines evidence-informed modalities to offer training, classroom consultation, intensive intervention, and peer mentoring for parents and teachers in an integrated model. Blending it in the child’s natural environment increases the overall efficiency and opportunity for an enduring solution.

## References

[CR1] Achenbach, T., & Rescorla, L. (2000). An integrated system of multi-informant assessment. Excerpt from Manual for the ASEBA Preschool Forms & Profiles. Burlington, VT: University of Vermont, Research Center for Children, Youth, & Families, pp. 74–100. Retrieved from http://www.aseba.org/ordering/ASEBA%20Reliability%20&%20Validity-Pre-school%20.pdf.

[CR2] Amaya-Jackson L, DeRosa R (2007). Treatment considerations for clinicians in applying evidence-based practice to complex presentations in child trauma. Journal of Traumatic Stress.

[CR3] Arvidson J, Kinniburgh K, Howard K, Spinazzola J, Strothers H, Evans M, Blaustein M (2011). Treatment of complex trauma in young children: Developmental and cultural considerations in application of the ARC intervention model. Journal of Child & Adolescent Trauma.

[CR4] Blaustein M, Kinniburgh K (2010). Treating traumatic stress in children and adolescents: How to foster resilience through attachment, self-regulation, and competency.

[CR5] Blodgett, C. (2012). Adopting ACES screening and assessment in child serving systems. Working paper. http://extension.wsu.edu/ahec/trauma/Documents/ACE%20Screening%20and%20Assessment%20in%20Child%20Serving%20Systems%207-12%20final.pdf.

[CR6] Bratton S, Ceballos P, Sheely-Moore A, Meany-Walen K, Pronchenko Y, Jones L (2012). Head Start early mental health intervention: Effects of child-centered play therapy on disruptive behaviors. International Journal of Play Therapy.

[CR7] Briggs-Gowan MJ, Ford JD, Fraleigh L, McCarthy K, Carter AS (2010). Prevalence of exposure to potentially traumatic events in a healthy birth cohort of very young children in the northeastern United States. Journal of Traumatic Stress.

[CR8] Cohen J, Mannarino A, Deblinger E (2006). Treating trauma and traumatic grief in children and adolescents.

[CR9] Cook A, Spinazzola J, Ford J, Lanktree C, Blaustein M, Cloitre M, van der Kolk B (2005). Complex trauma in children and adolescents. Psychiatric Annals.

[CR10] Felitti V, Anda R, Nordenberg D, Williamson D, Spitz A, Edwards V, Marks J (1998). Relationship of childhood abuse and household dysfunction to many of the leading causes of death in adults: The Adverse Childhood Experiences (ACE) Study. American Journal of Preventive Medicine.

[CR11] Finkelhor D, Turner H, Ormond R, Hamby S (2009). Violence, abuse, and crime exposure in a national sample of children and youth. Pediatrics.

[CR12] Garro A, Brandwein D, Calafiore T, Rittenhouse N (2011). Understanding and addressing early childhood trauma. Communique.

[CR13] Ghosh Ippen C, Harris W, Van Horn P, Lieberman A (2011). Traumatic and stressful events in early childhood: Can treatment help those at highest risk?. Child Abuse and Neglect.

[CR14] Gilliam W (2005). Prekindergarteners left behind: Expulsion rates in state prekindergarten systems.

[CR15] Graham-Bermann S, Castor L, Miller L, Howell K (2012). The impact of intimate partner violence and additional traumatic events on trauma symptoms and PTSD in preschool-aged children. Journal of Traumatic Stress.

[CR16] Lieberman A, Chu A, Van Horn P, Harris W (2011). Trauma in early childhood: Empirical evidence and clinical implications. Development and Psychopathology.

[CR17] Lieberman A, VanHorn P (2009). Giving voice to the unsayable: Repairing the effects of trauma in infancy and early childhood. Child and Adolescent Psychiatric Clinics of North America.

[CR18] Olafson E, Connelly L, Sperry L (2012). Child abuse assessment strategy and inventories. Family assessment: Contemporary and cutting-edge strategies.

[CR19] Osofsky JD, Lieberman AF (2011). A call for integrating a mental health perspective into systems of care for abused and neglected infants and young children. American Psychologist.

[CR20] Perry D, Dallas Allen M, Brennan E, Bradley J (2010). The evidence base for mental health consultation in early childhood settings: A research synthesis addressing children’s behavioral outcomes. Early Education & Development.

[CR21] Resource Development Institute (2012). Head Start Trauma Smart 2011–2012 evaluation report.

[CR22] Resource Development Institute (2012). Head Start Trauma Smart evaluation report.

[CR23] Scheeringa M, Salloum A, Arnberger R, Weems C, Amaya-Jackson L, Cohen J (2007). Feasibility and effectiveness of cognitive-behavioral therapy for posttraumatic stress disorder in preschool children: Two case reports. Journal of Traumatic Stress.

[CR24] Shahinfar A, Fox N, Leavitt L (2000). Preschool children’s exposure to violence: Relation of behavior problems to parent and child reports. American Journal of Orthopsychiatry.

[CR25] Silverman WK, Ortiz CD, Viswesvaran C, Burns BJ, Kolko DJ, Putnam FW, Amaya-Jackson L (2008). Evidence-based psychosocial treatments for children and adolescents exposed to traumatic events. Journal of Clinical Child & Adolescent Psychology.

[CR26] Spinazzola J, Ford J, van der Zucker M, Kolk B, Silva S, Smith S, Blaustein M (2005). Survey evaluates complex trauma exposure, outcome, and intervention among children and adolescents. Psychiatric Annals.

[CR27] Stallard P (2006). Psychological interventions for post-traumatic reactions in children and young people: A review of randomised controlled trials. Clinical Psychology Review.

[CR28] Stein B, Jaycox L, Kataoka S, Wong M, Tu W, Elliott M, Fink A (2003). A mental health intervention schoolchildren exposed to violence: A randomized controlled trial. Journal of the American Medical Association.

[CR29] Swick K, Knopf H, Williams R, Fields M (2013). Family-school strategies for responding to the needs of children experiencing chronic stress. Early Childhood Education Journal.

[CR30] Teachstone. (n.d.). *Summary of Toddler and Pre-K CLASS Research.* Retrieved from http://www.teachstone.org/wp-content/uploads/2012/07/Toddler-Pre-K-CLASS-Research-Abstracts.pdf.

